# Solventless Conducting Paste Based on Graphene Nanoplatelets for Printing of Flexible, Standalone Routes in Room Temperature

**DOI:** 10.3390/nano8100829

**Published:** 2018-10-13

**Authors:** Andrzej Pepłowski, Piotr A. Walter, Daniel Janczak, Żaneta Górecka, Wojciech Święszkowski, Małgorzata Jakubowska

**Affiliations:** 1 Department of Microtechnology and Nanotechnology, Faculty of Mechatronics, Warsaw University of Technology, 8 A. Boboli St., 02-525 Warsaw, Poland; p.walter@mchtr.pw.edu.pl (P.A.W.); d.Janczak@mchtr.pw.edu.pl (D.J.); maljakub@mchtr.pw.edu.pl (M.J.); 2 Faculty of Material Science and Engineering, Warsaw University of Technology, 141 Wołoska St., 02-507 Warsaw, Poland; zaneta.gorecka.dokt@pw.edu.pl (Ż.G.); wojciech.swieszkowski@pw.edu.pl (W.Ś.)

**Keywords:** printed electronics, graphene composite, graphene nanoplatelets, flexible electronics

## Abstract

Novel printable composites based on high aspect ratio graphene nanoplatelets (GNPs), fabricated without using solvents, and at room temperature, that can be employed for flexible, standalone conducting lines for wearable electronics are presented. The percolation threshold of examined composites was determined to be as low as 0.147 vol% content of GNPs. Obtained sheet resistance values were as low as 6.1 Ω/sq. Stretching and bending tests are presented, proving suitability of the composite for flexible applications as the composite retains its conductivity even after 180° folding and 13.5% elongation.

## 1. Introduction

The printing of electronic devices is a branch of technology intensively developed both by research institutions and industry. This method of fabrication allows easy processing of materials without rigorous restrictions for the production lines such as clean rooms, protective atmospheres, etc. Researchers have focused on flexible application with employment of printing substrates such as polymer foils [1], paper [2] or even fabrics [3]. Numerous printing techniques, such as screen printing [4], flexography [5] or ink-jet printing [6], have been present for a long time in the printed electronics industry. With the use of functional materials such as microscale and nanoscale metal particles, various carbon allotropes or precisely engineered nanocomposites, printed layers for numerous applications can be fabricated, e.g., conductive [7,8], resistive [9,10], dielectric [11,12] or sensitive layers [13,14].

In numerous wearable applications, which are a constantly growing market [15], novel biosensors are introduced [16] with a significant contribution of printed sensors [17]. In these devices, biological components such as enzymes [18], nucleic acids [19], anti-bodies [20] etc., play crucial roles as bio-recognition agents, allowing detection of desired biological analytes such as hormones [21] or metabolites [22,23]. Given the vast range of these bio-recognition agents, incorporating them into printed structures is a demanding task, which often involves multiple modification steps and thus elongates fabrication time of the final device. It creates a set of restrictions for printing technology, such as low temperature (below 40 °C) curing, meeting biocompatibility with a given compound, etc. In this work we present printing paste that is cured in room temperature and does not involve any solvents, which, together with the non-toxicity of the silicone vehicle, eliminates the risk of disrupting biological particles. This potential platform for various biomedical applications was examined mainly for its electrical properties. Thus, enabling future employment of the material in applications requiring conductive, resistive as well as dielectric properties. Flexibility of the composite was also briefly examined, proving that it is suitable for printed wearable devices. As filler material for prepared composite, graphene nanoplatelets (GNPs) with a high aspect ratio were employed, as they were previously confirmed to contribute to performance of voltammetric and potentiometric sensors [24,25]. It was also proved that a high aspect ratio enables lower percolation thresholds [26], thus lowering the required amount of the functional phase.

## 2. Materials and Methods

### 2.1. Materials

Graphene nanoplatelets type M15 and M25 (thickness 6 nm, medium diameter 15 µm and 25 µm, respectively) were purchased from XG Sciences (Lansing, MI, USA, USA). Silver microflakes-based L-121 paste was delivered by Institute of Electronic Materials Technology (ITME, Warsaw, Poland). 3140-RTV silicone rubber (RTV) was acquired from Farnell Ltd. (Leeds, UK). Dispersing agent AKM-0531 was purchased from NOF Corp. (Tokyo, Japan). Poly (ethylene terephthalate) (PET) foil Melinex 453 with a thickness of 100 µm was delivered by TEKRA (Milwaukee, WI, USA).

### 2.2. Preparation of Printed Layers

Prior to addition to silicone vehicle, GNPs were sonicated in acetone for 1 hour with an addition of 3 wt% dispersing agent to disperse any agglomerates and dried for 30 min. at 150 °C. After deagglomeration of nanoplatelets, they were added to silicone rubber in the proportions given in Table 1, and mixed using an agate mortar until uniform filler distribution was achieved.

**Figure 1:**
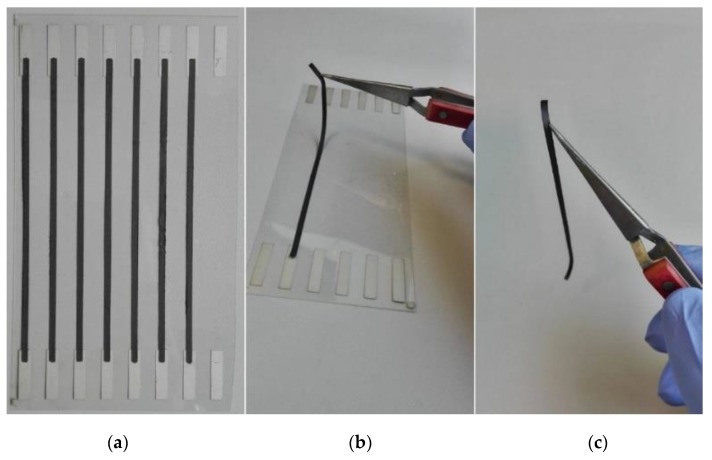
Conductive routes printed with silicone/graphene nanoplatelets composite. (**a**) samples with silver electrical contacts; (**b**) detachment of the sample from substrate; (**c**) standalone conducting route.

Employing silver microflake-based polymer paste, electrical contacts were printed on PET foil using a SPP-600FV screen-printer (Tampoexpert s.c., Warsaw, Poland) with 68T polyester screens. The printed layer was cured at 120 °C for 30 min. Then, overlapping on the electrical contacts, conducting routes were printed with prepared graphene/silicone composite pastes using stencils cut in PET foil. Dimensions of the printed routes: length *L* = 110 mm, width *W* = 2 mm, layer thickness *t =* 100 µm. Routes were kept in room temperature for 24 hours to allow full cross-linking of the composite [27]. Since the paste contains no solvent, composition of the samples before and after cross-linking were assumed to be equal. A set of printed samples is shown in Figure 1a. Due to the composite flexibility, it was possible to detach conducting routes from the substrate (Figure 1b,c) end examine their properties under mechanical deformation.

### 2.3. Characterization

**Figure 2:**
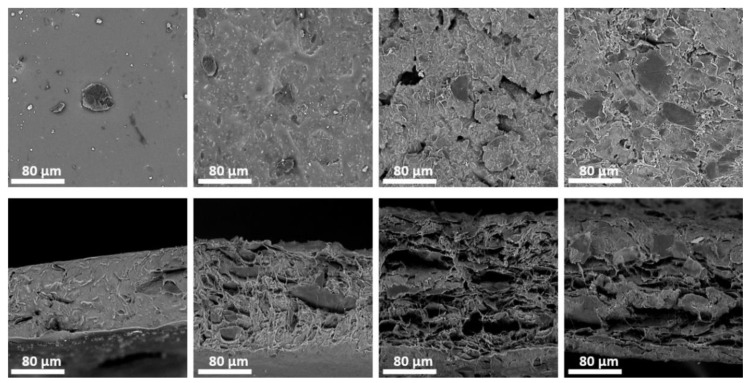
SEM micrographs of prepared composite layers, from left to right: 8, 15, 27 and 45 wt% graphene nanoplatelets content. Top: surface of the samples; on the surface of 8 wt% sample only single GNPs were visible, for 15 wt% outlines are visible from below silicone, for 27 and 45 wt% samples only a thin layer of silicone covers otherwise visible graphene structure. Bottom: cross-sections; for 8 wt% sample close to homogenous silicone structure is visible, which corresponds to observed insulating properties of the samples below 9 wt% graphene content.

Scanning electron microscopy (SEM) was done using Phenom ProX (Phenom-World, Eindhoven, The Netherlands). Observations were carried out with magnification ×1000. Acceleration voltage was 10 kV. An integrated backscattered electron detector (full mode) was used in SEM analysis. The detector used in the Phenom microscope provides material contrast and topographic imaging in parallel with what was useful in the analysis of measured samples. Cross-sections were obtained by cutting composites with scissors in room temperature. Both top and cross-sectional micrographs are shown in Figure 2.

Electrical resistance (Table 1) of the conducting routes was measured through a four-point method employing Keysight 34461A (Keysight Technologies, Santa Rosa, CA, USA) multimeter. Sheet resistance R_S_ was calculated from measured values and sample dimensions (Equation (1), *R* = measured resistance value, *W* = layer’s width, *L* = length of the layer). Next, electrical conductivity was calculated as reciprocal of resistivity ρ in Equation (2), where t -layer’s thickness. For values of *W*, *L*, *t*, see Section 2.2.

(1) $$R_{S}=R\cdot \frac{W}{L}$$

(2) $$\rho =R_{S}\cdot t$$

## 3. Results and Discussion

### 3.1. Experimental Determination of Conductivity Threshold

**Figure 3:**
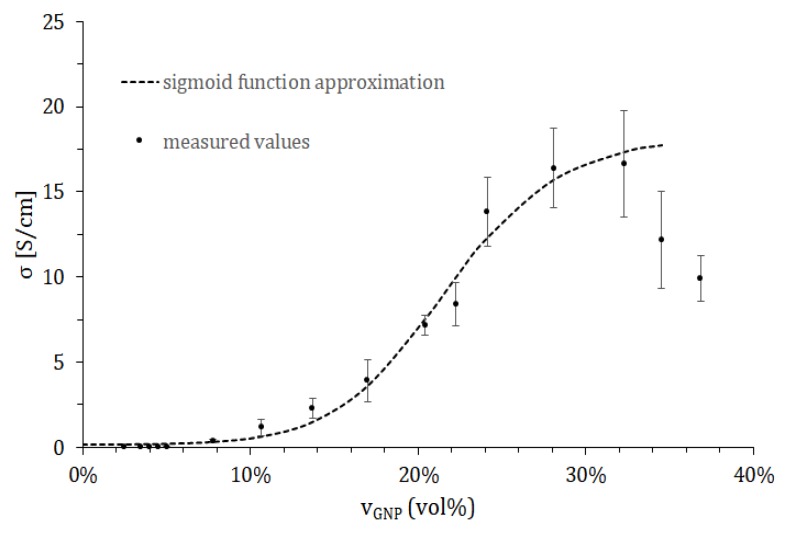
Electrical conductivity of silicone/graphene nanoplatelets composite depending on volumetric content of the graphene with fitted sigmoid function (Equation (3) and Table 2).

**Figure 4:**
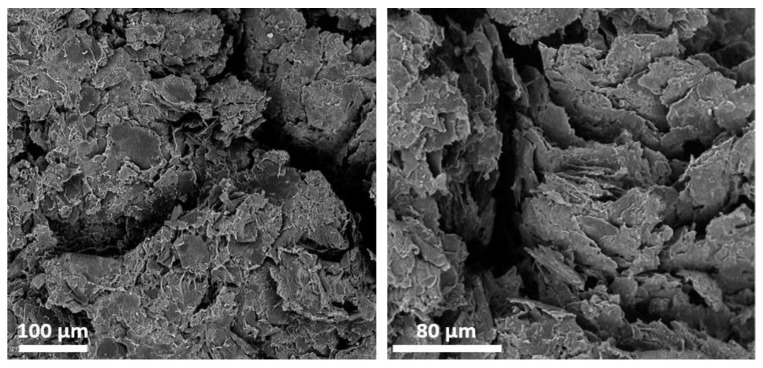
SEM images of composite fractures for 55 wt% (36.8 vol%) GNP sample.

Electrical resistance of the samples prepared as described in Section 2.2 was measured. Obtained values allowed computing of the composites’ sheet resistance (Table 1) and electrical conductivity (Figure 3). For samples below 9 wt% GNP content, measured resistance values exceeded measurement range, i.e., 40 MΩ (data shown only for 8 wt% sample). Conversion from wt% to vol% content of GNP was calculated assuming density of GNP $\rho_{GNP}=2.2\;g/{\mathrm{cm}}^{3}$, being equal to the density of graphite [28], given the same crystal structure of both materials. Thus, 9 wt% content of GNP corresponds to 4.51 vol% and can be interpreted as the lower limit of filler concentration allowing electrical conductivity in most applications ($v_{EX}$). This determination, however, is only coarse and prone to deviation, therefore theoretical models of percolation were employed for precise results. On the other hand, conductivity of the samples with GNP content above 45 wt% was not further increasing, dropping drastically above 50 wt%. It was presumed that this effect was caused by increased brittleness of the composite, which was confirmed by SEM imaging (Figure 4).

As the first approximation, a sigmoid function (Figure 3) was fitted to experimental data for characterization of both lower and upper conductivity plateaus. The function fitted was in accordance with Equation (3). For fitting, Solver tool (Frontline Systems Inc., Incline Village, NV, USA) was employed with mean square error criterion. In computation, points beyond *v_GNP_* = 32 vol% were not included, since conductivity of composites was disturbed by their brittleness. In Table 2. the fitted function parameters are listed.

(3) $$\sigma \left(v_{GNP}\right)=\;\frac{a}{b+c\cdot e^{-d\left(v_{GNP}-f\right)}}+t$$

### 3.2. Classical Percolation Theory

According to percolation theory described in 1991 by Stauffer and Aharony [29], electrical conductivity of a composite comprised of an insulating matrix and conductive filler can be described as in Equation (3) below, where: $\sigma_{c}$ = conductivity of the composite, $\sigma_{GNP}$ = conductivity of filler (in this case: graphene nanoplatelets), $v_{GNP}$ = volumetric content of the filler, $v_{p}$ = percolation threshold and *β* = critical exponent (2 for randomly distributed resistor model in Reference [29]).

(4) $$\sigma_{c}=\sigma_{GNP}{\left(v_{GNP}-v_{p}\right)}^{\beta }$$

First, single GNP particle conductivity was calculated, as it is known that multi-layer graphene-like structures exhibit different physical properties depending on layer number and geometrical dimensions [30]. As specified by the manufacturer [31], a single GNP particle falls within the range of 6–8 nm. According to work by Romanenko et al. [32], it corresponds to 19 layers of graphene. Thus, conductivity of a single M25 GNP can be approximated as $\sigma_{GNP}\approx 6.23\times 10^{4}S/\mathrm{cm}$.

**Figure 5:**
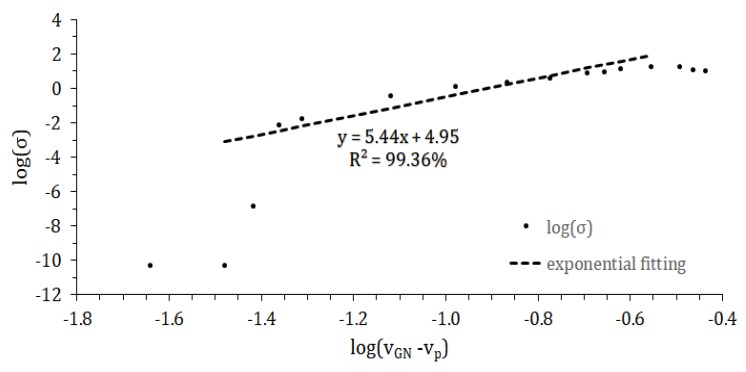
Linear fitting of silicone/graphene nanoplatelets composite conductivity logarithm versus logarithm of graphene volumetric fraction. Points under 8 vol% and above 25 vol% content were not included due to conductivity plateau.

Fitting of the critical exponent (shown in Figure 5) yielded β≈5.44. Substituting this value and solving Equation (3) with respect to percolation threshold yields $v_{p}\approx 0.167$ vol%, which is well below the one observed as conductivity threshold (4.51 vol%). This significant difference could be explained based on the statistical nature of percolation. As given in Table 1, standard deviation of *R_S_* for samples below 15 wt% GNP equals more than half of the mean value. This was interpreted as an indication of a low probability of forming conducting lines in composite. In addition, there is an assumption that above $v_{p}$ at least one conducting line is formed, which may result in conductivity below values measurable by apparatus. For verification of this interpretation, two other models of percolation were employed.

### 3.3. Comparison with Other Approaches

As shown in the work of Balberg et al. [33], certain volumes can be defined around filler particles, into which the center of another particle is not allowed to enter (excluded volume). It can be shown that overlapping of excluded volumes is sufficient to form a conducting link [34]. Thus, percolation threshold can be expressed in relation to the excluded volume coefficient ($V_{ex}$, depending on the shape of particle) and particle specific dimensions (in the case of GNPs: *d* = thickness, *r* = radius) as follows:

(5) $$v_{p}=1-\;e^{-\frac{V_{ex}d}{\pi r}}$$

From work by Charlaix [35], for particle shaped as an infinitely thin disc $V_{ex}$ assumes value of $V_{ex}=1.8$. Analogically, for spherical particles $V_{ex}=2.8$ as described by Celzard et al. [36]. Since GNPs have non-negligible thickness, $V_{ex}$ is predicted to lie between those two numbers. Thus, the following double inequality for $v_{p}$ can be formulated:

(6) $$1-\;e^{-1.8d/\pi r}\leq \;v_{p}\;\leq 1-\;e^{-3.8d/\pi r}$$

The mean radius of GNPs was assumed to be 12.5 µm, according to the material supplier’s information. The critical distance between two particles, below which the tunneling effect occurs, allowing electric conductivity, is approx. 10 nm [37]. Hence, thickness of the GNPs was assumed as 6 nm, given by the manufacturer, plus ±10 nm range of tunneling effect, i.e., 26 nm in total. Substituting all of the above into Equation (5), the following range was obtained:

(7) $$0.119\mathrm{vol}\%\leq \;v_{p}\;\leq 0.185\mathrm{vol}\%$$

This is in accordance with findings by Wu et al. [26], and with results obtained from the Stauffer/Aharony model. Next, the model described by Helsing and Helte [38] as mean field theory was employed. In this model, the polymer matrix and conductive filler structure are approximated as a homogenous system, which represents a random resistor network. Derived from this assumption, an empirical formula for the percolation threshold is given:

(8) $$v_{p}=1.18\eta$$

where η is filler particle aspect ratio—in this case: Thickness divided by diameter. Substituting this dimension (including the 20 nm thickness margin mentioned above), Equation (8) yields $v_{p}\approx 0.123\;\mathrm{vol}\%$, which corresponds to the results obtained from previous calculations.

Summarizing these considerations, a set of volumetric fractions is obtained, corresponding to the onset of electrical conductivity in RTV/GNP composite. These values are listed in Table 3, and since they are within a range of ± 0.025 vol% from the mean, it can be concluded that the results were coherent. Thus, mean value $v_{p}\approx 0.147\;\mathrm{vol}\%$, can be named composite’s percolation threshold.

### 3.4. Flexibility Tests

**Figure 6:**
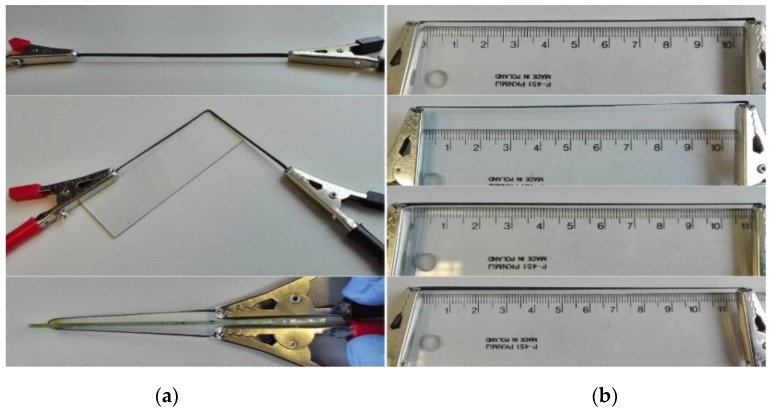
Procedures employed for flexibility testing of RTV/GNP composite testing: (**a**) – bending, angle, from top to bottom: 0°, 90°, 180°; (**b**) – stretching, elongation, from top to bottom: 0%, 4.5%, 9%, 13.5% (glass and ruler are shown for scale only).

For assessment of the RTV/GNP composite applicability in wearable and flexible electronics, two tests were employed: Bending and stretching (Figure 6). Experimentally, a 15 wt% sample was selected, as it exhibited the highest endurance to deformation (data not shown). Results of the tests, and values of the sample resistance are listed in Table 4.

Bending of the sample firstly resulted in a decrease of resistance value by 16% (90°), but further deformation (180°) caused only 1% increase. Initial resistance drop was caused by local compression of the composite structure in the inner plane of bending. Simultaneously, the outer plane was subject to stretching, which counterbalanced further resistance decrease. During longitudinal stretching of the sample, close to exponential ($R^{2}=94.01\%$, data not shown) increase of resistance was observed. Above 13.5% elongation, visible disruption of the sample structure was observed, hence further results are not given. Below this level, however, resistance of the sample was observed to return over time to the initial value, which indicates that the composite structure was intact.

## 4. Conclusions

A conductive composite is presented with low percolation threshold $v_{p}\approx 0.147\;\mathrm{vol}\%$, which is consistent with other reports [26,39] concerning GNP-based materials. Concentration of graphene for practical application in conducting structures was also found, which will enable development of electrodes for various biosensing techniques. Flexibility of the composite was also tested, proving that the material retains its conductive properties after bending up to 180°, as well as after longitudinal stretching up to 13.5%. It is crucial for applying printed layers for wearable devices and will be further investigated for optimal composition.

## Figures and Tables

**Table 1:** Proportions for prepared RTV/GNP pastes and respective sheet resistance values (R_S_), errors are one standard deviation (not given for samples below 9 wt% due to route resistance exceeding measurement range); for samples below 9 wt% GNP, measured resistance exceeded ohmmeter upper limit of measurable resistance, so it was assumed as the lower limit of sample resistance, upper limit is derived from the bare silicone characteristics [27].

Sample – GNP Content (wt%)	R_S_ (Ω/sq)
GNP-5%	7.27 × 10^5^ ÷ 2.1 × 10^12^
GNP-7%	7.27 × 10^5^ ÷ 2.1 × 10^12^
GNP-8%	7.27 × 10^5^ ÷ 2.1 × 10^12^
GNP-9%	1.7 × 10^4^ ± 1.1 × 10^4^
GNP-10%	8.1 × 10^3^ ± 5.1 × 10^3^
GNP-15%	287 ± 69
GNP-20%	101 ± 41
GNP-25%	47 ± 12
GNP-30%	28.6 ± 9.0
GNP-35%	36.3 ± 6.3
GNP-37.5%	11.9 ± 0.4
GNP-40%	7.2 ± 0.6
GNP-45%	6.2 ± 0.9
GNP-50%	6.1 ± 1.2
GNP-52.5%	8.9 ± 2.1
GNP-55%	10.5 ± 1.4

**Table 2:** Parameters of sigmoid function fitted to experimental conductivity data, parameters corresponding to Equation (3).

a	b	c	d	f	t
2.328524	0.127924	0.154164	21.55641	0.358685	0.158932

**Table 3:** Volumetric content of graphene nanoplatelets in RTV/GNP composite corresponding to the onset of conductivity: $v_{EX}$ = critical fraction observed during experiment, $v_{CP}$ = percolation threshold according to classical percolation equation [28], $v_{EV}$ = percolation threshold range in excluded volume approach [30], $v_{MF}$ = percolation threshold derived from mean field theory [31].

$v_{EX}\;(\mathrm{vol}\%)$	$v_{CP}\;(\mathrm{vol}\%)$	$v_{EV}\;(\mathrm{vol}\%)$	$v_{MF}\;(\mathrm{vol}\%)$
4.51	0.167	〈0.119 ÷0.185〉	0.123

**Table 4:** Resistance of a 15 wt% RTV/GNP composite route with length of 110 mm and width of 2 mm during bending and stretching as shown in Figure 6.

**Bending**	angle (°)	0	90	180		
	*R* (kΩ)	43.9	36.9	37.3		
**Stretching**	elongation (%)	0	4.5	9	13.5	
	*R* (kΩ)	43.9	68	307	1.6 × 10^4^	
